# Supplementary material for: A promising material for bone repair: PMMA bone cement modified by dopamine-coated strontium-doped calcium polyphosphate particles
Source: R Soc Open Sci. 2019 Oct 2;6(10):191028. doi: 10.1098/rsos.191028 (PMC6837193; doi:10.1098/rsos.191028)
Supplement: Supplementary Experimental method and results [file rsos191028supp1.docx]

**Supplementary material**

**Supplementary Experimental method**

Preparation of SCPP/PMMA and D/SCPP/PMMA composite bone cements

Characterization of pore size distribution and porosity of PMMA composite bone cements

Dispersion of SCPP and D/SCPP particles in PMMA matrix

Dough time test

Intrusion test

Setting time and maximum temperature test

Biomechanical properties test

In vitro mineralization performance of bone cement

MTT test

SEM observation

Immunofluorescence staining

ELISA measurement

**Supplementary Results**

Detection of Sr and DOPA in PMMA Composites

Supplementary Figure 1 EDS test and elemental mass percentage of PMMA bone cement composite

Supplementary Figure 2 XPS full spectrum and N peak of D/SCPP/PMMA

Supplementary Figure 3 Section of SEM of PMMA composite bone cement

Supplementary Figure 4 SEM analysis of PMMA,SCPP/PMMA,D/SCPP/PMMA after soaking in SBF for 6 days

Supplementary Figure 5 Comparison of the contents of Ca and P in the surface-precipitated layer of PMMA,SCPP/PMMA,D/SCPP/PMMA after soaking in SBF for 6 days

Supplementary Figure 6 The SEM images of adhesion and growth of MG63 cells on PMMA composite bone cement

Supplementary Table 1 The composition of D/SCPP/PMMA

Supplementary Table 2 Pore volume and pore size of SCPP/PMMA and D/SCPP/PMMA

Supplementary Table 3 The contents of Ca and P in the surface-precipitated layer of PMMA,SCPP/PMMA,D/SCPP/PMMA after soaking in SBF for 6 days

**Supplementary Experimental method**

**Preparation of SCPP/PMMA and D/SCPP/PMMA composite bone cements**

8% β-SCPP particles with an average size of 100 μm were prepared by the previous method. Meanwhile, a 2.0 mg per mL DOPA solution was prepared by dissolving dopamine (Sigma Company, USA) in Tris-HCl buffer solution (10 mM, PH 8.5). 8% β-SCPP particles were immersed in an excess amount of 2 mg/ml DOPA solution for 24 hours with continuous shaking. Subsequently, specimens were taken out and rinsed with deionized water to remove physically adsorbed DOPA, and then were dried in an oven at 37° C to obtain DOPA-coated 8% β-SCPP particles. Then, the SCPP particles and D/SCPP particles were admixed to pre-polymerized beads PMMA.

The weight fractions of the D/SCPP particles in D/SCPP/PMMA composite bone cements were shown in Supplementary Table 1. Similar weight fractions of the SCPP particles were also applied in SCPP/PMMA composite bone cements. The pure PMMA bone cements served as a control group. The bone cement pastes were added to a 75×10×3.3 mm rectangular mold to fabricate rectangular samples for flexural bending tests. They were also respectively added to a Φ 6×12 mm and Φ10×2 mm cylindrical mold to fabricate cylindrical samples for compression experiments and biological experiments.

**Detection of Sr and DOPA in PMMA composite bone cements**

The XSAM 800 XPS analyzer (Kratos) was used to detect the elements only present in DOPA and SCPP. The EDS was used to test the chemical composition of D/SCPP/PMMA and SCPP/PMMA composite bone cement material.

**Characterization of pore size distribution and porosity of PMMA composite bone cements**

The cross-section morphology of the PMMA composite bone cement was observed by a JSM-5900 LV scanning electron microscope (SEM), and the pore size distribution as well as porosity of PMMA composite bone cement were measured using a Tristar II 3020M automatic specific surface and pore analyzer (USA, Micromeritics Instrument Corporation)

**Dispersion of SCPP and D/SCPP particles in PMMA matrix**

Micro-CT analysis was performed on cylindrical samples of Φ6×12mm PMMA, SCPP/PMMA, and D/SCPP/PMMA composite cements using a VivaCT 80 (SCANCO Medical AG) microscopic imaging system to confirm the dispersion of SCPP and D/SCPP particles throughout the PMMA matrix.

**Dough time test**

Dough time is defined as the moment when the cement should be applied. Testing was carried out according to ISO 5833 and ASTM F-451 test methods. At 23 ± 1°C ambient conditions, the D/SCPP particles-PMMA beads mixer or SCPP particles-PMMA beads mixer was dissolved in MMA monomer using solid: liquid ratio given in Table 1 and the mixing started. After mixing for 1 min, a finger wearing a latex glove probed the fresh surface of the paste every 20 s until the gloved finger separated cleanly from the surface of the paste. This time was recorded as the dough time of the cement. All the tests were performed in triplicate (n= 3).

**Intrusion test**

At 23±1°C ambient conditions, the mixer mentioned above was admixed to MMA monomer according to the ratio in Table 1. After reaching the dough time for 1 min, the paste was intruded into a hole with 10mm in depth and 1mm in diameter on the cylinder type intrusion mold with one end open with a force of 49 N. This process lasted for 1 min. The length of cement intruded into the mold is the intrusion of bone cement. All the tests were performed in triplicate (n= 3).

**Setting time and maximum temperature test**

At 23±1°C ambient conditions, the cement prepared above was filled into a Teflon mold (6mm height, 60mm diameter) and the temperature was measured by a type K thermocouple (Testo 925, Lenzkirch, Germany). The probe of the thermocouple was put at the center of the paste surface and the maximum temperature (Tmax) during the polymerization reaction was recorded. As for the setting time, it was defined as the time corresponding to the average value of the maximum and the ambient temperature.

**Biomechanical properties test**

Biomechanical properties of the bone cements were tested on a universal material testing machine (Ag-10TA, USA) according to the ISO5833 standard. The samples for the compressive test were added to a Φ 6×12 mm cylindrical mould. The flexural bending tests, with a supporting span of 60mm, was measured using rectangular shaped samples (75×10×3.3 mm). All the tests were performed at a crosshead speed of 5 mm/min at room temperature. Five duplicates were measured for each sample.

**In vitro mineralization performance of bone cement**

Samples of the bone cement with Φ10×2mm were soaked in SBF at 37°C for 6 days for biomineralization. The samples were washed with distilled water and then dried at 23±1°C ambient conditions. The mineral layer on the surface of samples was characterized by XRD, SEM and EDS.

**MTT test**

The MTT assay was performed to assess cell proliferation.On the 1,4,7day,60uL per well of MTT solution(5 mg ml-1 in phosphate buffered saline,PS)was added and 4 h further incubation was carried out to crystallize formazan completely.Then the liquid in the well was sucked off,and 400 uL per well dimethylsulfoxide(DMSO) was added,followed by constant shaking for 10 min to dissolve formazan crystal completely.The optical density(OD) value of this solution was measured at 492 nm with a microplate reader(Model 550, Bio Rad Corp).

**SEM observation**

Cytocompatibility of MG63 cultured on the specimens was also studied by observing using SEM. After culturing for 4 days, the specimens with the cells were washed with PBS, and fixed in 3% glutaraldehyde at 4℃ overnight. The samples were then dehydrated in increasing the concentration of alcohol. The critical point drying of specimens was carried out with liquid CO_2_. The specimens were observed by SEM after their sputter-coating with gold.

**Immunofluorescence staining**

The bone cement samples with cells were washed twice in prewarmed PBS, fixed with 4% paraformaldehyde, and permeabilized with 0.1% Triton X-100 in PBS. And then, the F-actin and nucleus in cells were stained with Phalloidin and DAPI respectively and visualized by OLYMPUS U-HGLGPS. Phalloidin diluted 1:1000 was used for F-actin staining, DAPI diluted 1:100 was used for nuclear staining.

**ELISA measurement**

BMP-2,VEGF and ALP were determined by ELISA measurement. In tests, various bone cement specimens with seeded MG63 were prepared as described above. After 4 days co-culturing, supernatant liquid was collected through centrifuging at 14000rpm for 5min for ELISA measurement. A serious of standard curve for ALP, BMP-2 and VEGF were plotted using corresponding standard solution. After that, ELISA assay was carried out as Kit instruction (R&D Corp.), the concentration of ALP, BMP-2 and VEGF were accurately calculated and values were expressed as pg/ml.

**Supplementary Result**

**Detection of Sr and DOPA in PMMA Composites**

EDS test showed that the weight percentages (WP) and atomic percentages(AP) of Sr elements in SCPP/PMMA and D/SCPP/PMMA composite bone cements were 0.74%&0.12%, 0.44%&0.07%, respectively,which indicated that Sr elements had been incorporated into PMMA bone cements successfully. The WP and AP of N element in D/SCPP/PMMA material were 22.68% & 22.86% (Supplementary Figure 1), while the N 1s peak appears around 400eV in the XPS spectrum (Supplementary Figure 2), indicating the exist of -NH- functional group on DOPA, confirming successful coating of DOPA on SCPP surface and that D/SCPP particles had also been successfully incorporated into the PMMA bone cement matrix. Above characterizations of composite bone cement suggested that we obtained a novel PMMA composite bone cement successfully modified by dopamine-coated strontium-doped calcium polyphosphate Particles.


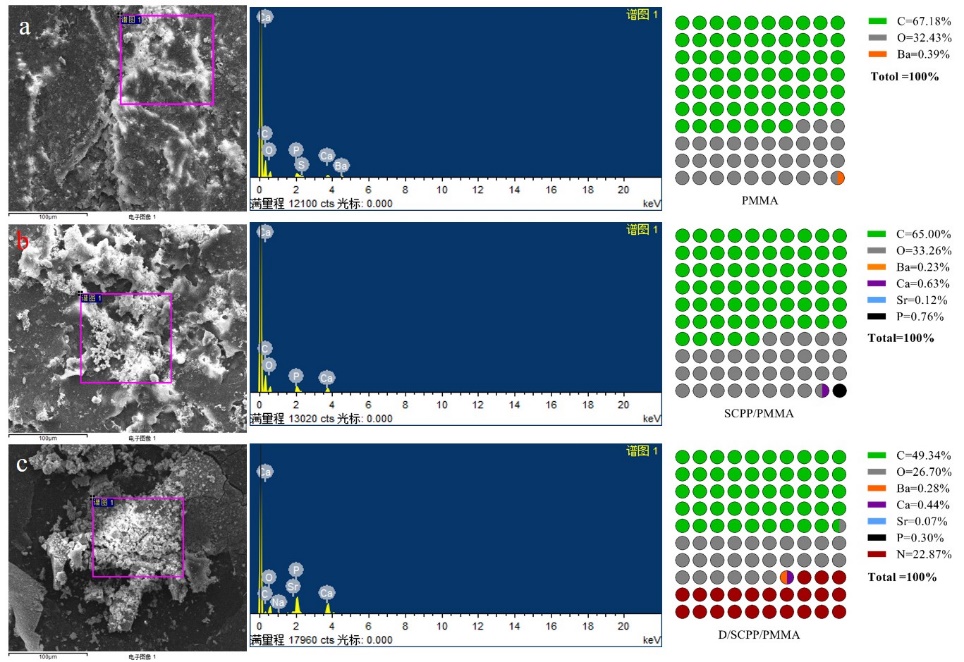


Supplementary Figure 1 EDS test and elemental mass percentage of PMMA bone cement composite.


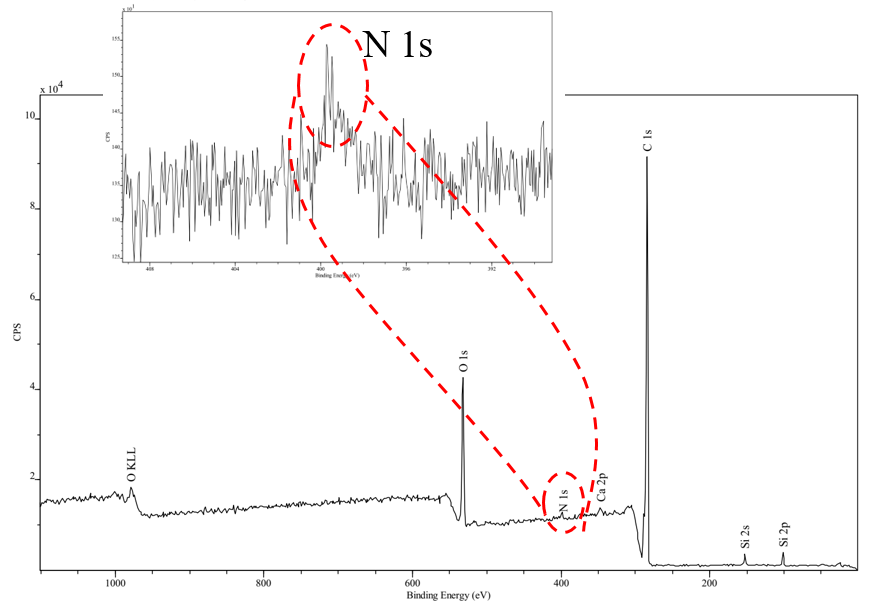


Supplementary Figure 2 XPS full spectrum and N peak of D/SCPP/PMMA


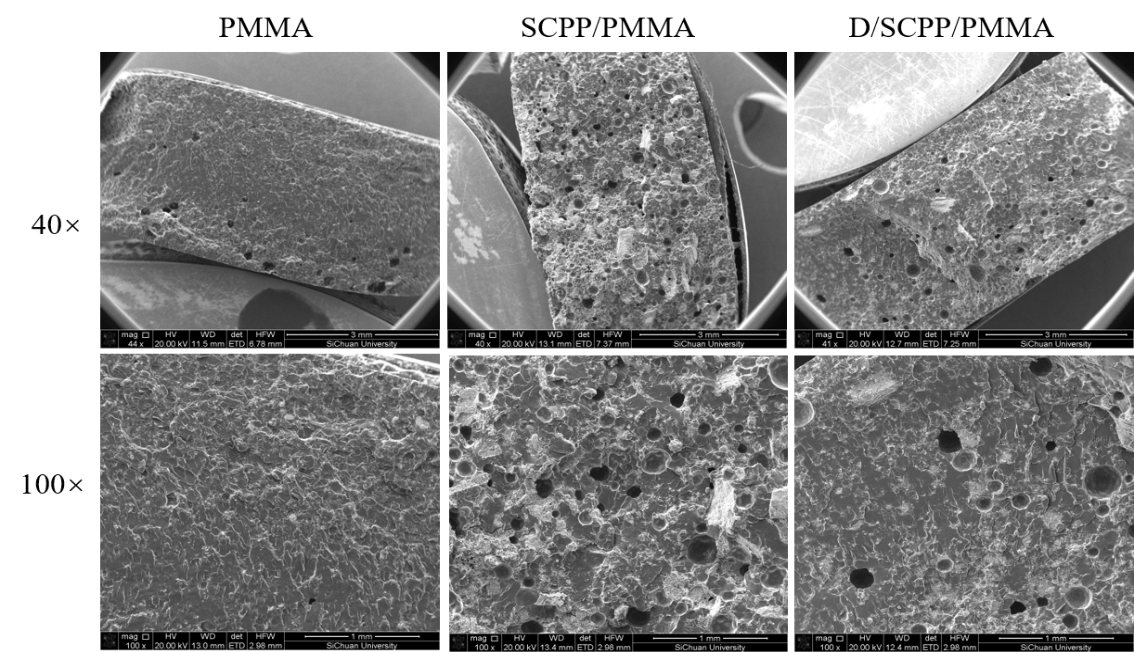


Supplementary Figure 3 Section of SEM of PMMA composite bone cement


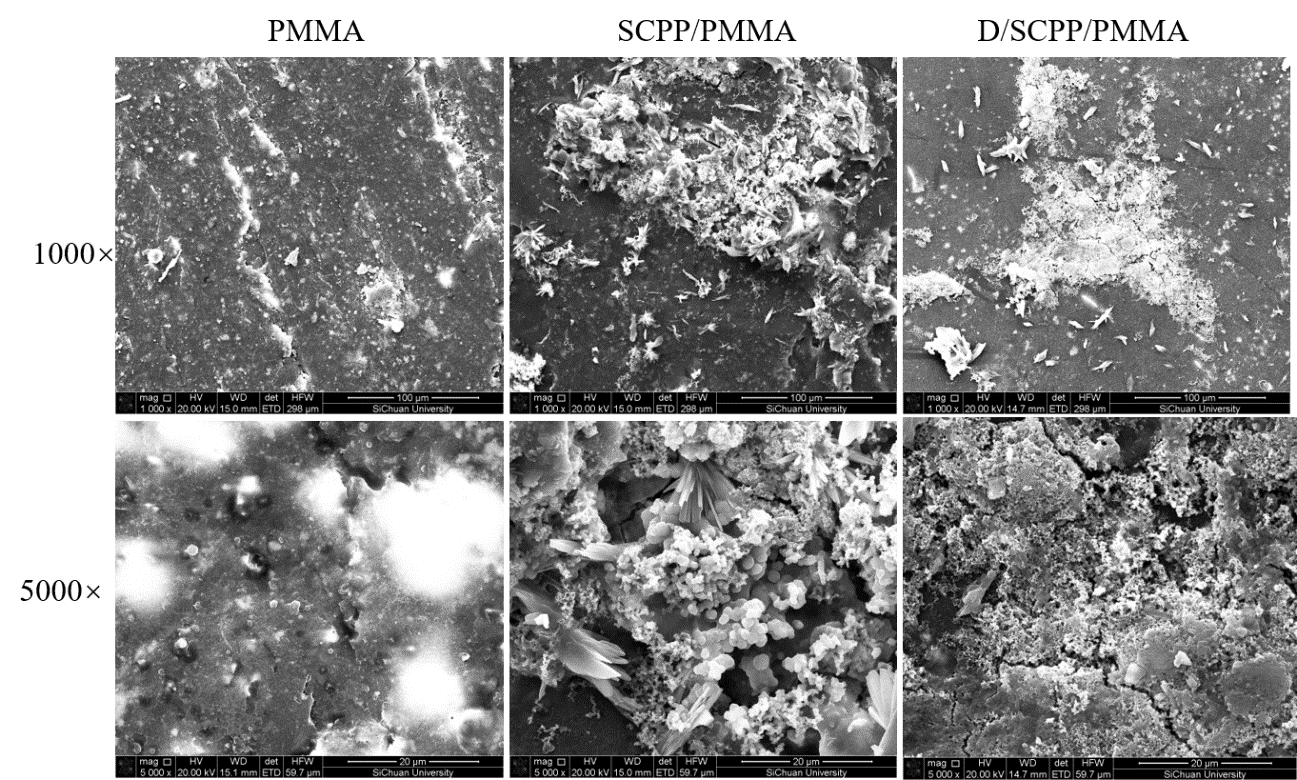


Supplementary Figure 4 SEM analysis of PMMA,SCPP/PMMA,D/SCPP/PMMA after soaking in SBF for 6 days


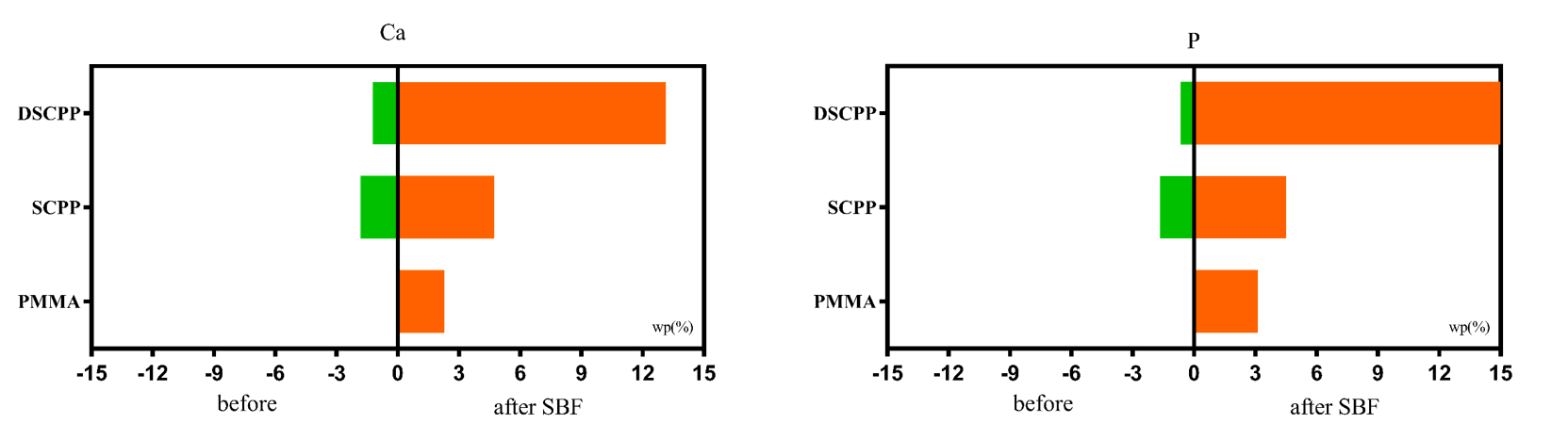


Supplementary Figure 5 Comparison of the contents of Ca and P in the surface-precipitated layer of PMMA,SCPP/PMMA,D/SCPP/PMMA after soaking in SBF for 6 days


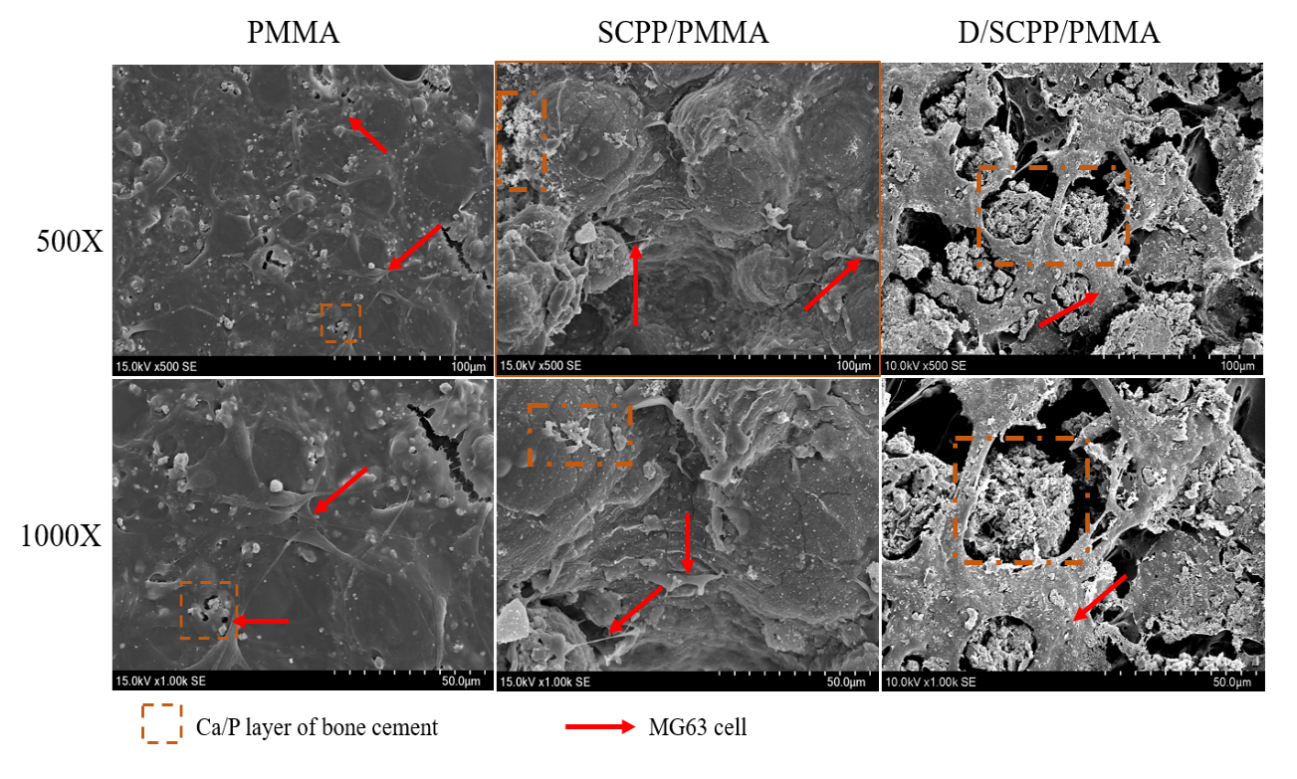


Supplementary Figure 6 The SEM images of adhesion and growth of MG63 cells on PMMA composite bone cement

**Supplementary Result**

Supplementary Table 1 The composition of D/SCPP/PMMA

| Bone cement | PMMA | 5%D/SCPP/ PMMA | 10%D/SCPP/ PMMA | 15%D/SCPP/  PMMA |
| --- | --- | --- | --- | --- |
| D/SCPP percentage(%)（wt%） | 0 | 5 | 10 | 15 |
| PMMA（g） | 2 | 1.9 | 1.8 | 1.7 |
| D/SCPP（g） | 0 | 0.1 | 0.2 | 0.3 |
| Liquid phase（ml） | 1 | 0.95 | 0.9 | 0.85 |
| Solid-liquid ratio | 2:1 | 2:0.95 | 2:0.9 | 2:0.85 |

Supplementary Table 2 Pore volume and pore size of SCPP/PMMA and D/SCPP/PMMA

| Test item | SCPP/PMMA | D/SCPP/PMMA |
| --- | --- | --- |
| Total pore volume  (p/p0=0.990)[cm3 g-1] | 0.7717 | 0.4591 |
| Average pore diameter [nm] | 3.5243 | 3.2511 |

Supplementary Table 3 The contents of Ca and P in the surface-precipitated layer of PMMA 、SCPP/PMMA、D/SCPP/PMMA after soaking in SBF for 6 days

| Element | PMMA | | SCPP/PMMA | | D/SCPP/PMMA | |
| --- | --- | --- | --- | --- | --- | --- |
|  | WP | AP | WP | AP | WP | AP |
| Ca | 2.29 | 0.82 | 4.73 | 1.65 | 13.14 | 5.87 |
| P | 3.13 | 1.44 | 4.51 | 2.04 | 15.47 | 8.94 |

WP：Weight Percentage; AP:Atom Percentage
